# Jiangzhi ruanmai recipe alleviates atherosclerosis in ApoE^−/−^ mice by regulating cholesterol metabolism involved in gut microbiota remodeling

**DOI:** 10.3389/fcvm.2026.1834983

**Published:** 2026-05-29

**Authors:** Guinan Xie, Lijun Zhang, An Yan, Guiyun Pan, Yudie Xu, Zhihui Song, Liping Guo, Yi Wang

**Affiliations:** 1 Institute of Traditional Chinese Medicine, Tianjin University of Traditional Chinese Medicine, Tianjin, China; 2 First Affiliated Hospital of Gannan Medical University, Ganzhou, Jiangxi, China; 3 Tianjin Academy of Traditional Chinese Medicine Affiliated Hospital, Tianjin, China; 4 Tianjin Academy of Traditional Chinese Medicine, Tianjin, China

**Keywords:** atherosclerosis, bile acids, cholesterol metabolism, cholesterol reverse transport, gut microbiota, gut microbiota-liver-cholesterol axis, Jiangzhi ruanmai recipe

## Abstract

**Background:**

Atherosclerosis is a public health problem with high morbidity and mortality,Gut microbiota dysbiosis disrupts bile acid metabolism, impedes cholesterol excretion, promotes arterial lipid deposition, and accelerates atherosclerosis. Jiangzhi ruanmai recipe (JZRM) is a Chinese herbal medicine prescribed for the treatment of hyperlipidemia and atherosclerosis. However, the mechanism is unclear.

**Purpose:**

This study aims to investigate the effects and mechanisms of JZRM on Atherosclerosis.

**Methods:**

LC-MS analysis was first performed to characterize the chemical composition of JZRM. Next, an atherosclerosis model was established in ApoE^−/−^ mice fed a high-fat diet for 16 weeks, and the therapeutic effects of JZRM were evaluated based on serum lipids, glucose, bile acids, and aortic plaque area. Fecal bile acid levels were subsequently measured, and 16S rRNA sequencing was employed to assess the abundance, diversity, and compositional changes of gut microbial communities. Finally, hepatic expression of genes and proteins involved in reverse cholesterol transport and bile acid synthesis was determined by qPCR and Western blot.

**Results:**

JZRM significantly reduced the lipid, glucose, and inflammation levels in mice; decreased the TBA levels of liver, intestine and faeces, and reduced the aortic plaque area; Second, JZRM increased the abundance and diversity of intestinal flora, significantly enhanced the level of intestinal bacteria that promote bile acid decomposition and excretion.Finally, JZRM significantly upregulated the protein expression of ABCA1, ABCG1, PPAR*γ*, and LXR*α*, as well as the mRNA expression of *Abca1*, *Abcg1*, *Pparg*, and *Nr1h3*.; In terms of bile acid metabolism, JZRM significantly regulates effective endogenous agonists chenodeoxycholic acid (CDCA) and lithocholic acid (LCA), as well as the FXR/CYP7A1 pathway to promote bile acid synthesis.

**Conclusion:**

JZRM regulates gut microbiota-liver-cholesterol axis, promotes cholesterol synthesis and metabolism to alleviate atherosclerosis.

## 1 Introduction

Atherosclerosis (AS) constitutes a critical pathophysiological basis for cardiovascular and cerebrovascular diseases and ranks among the leading causes of morbidity and mortality worldwide (1). It is characterized by thickening and hardening of the arterial wall, loss of elasticity, and narrowing of the lumen, as well as lipid accumulation on the arterial intima to form yellow atheromatous (2). When the atherosclerotic plaques severely block blood vessels, erode, or rupture, it is prone to cause acute cardiovascular and cerebrovascular events such as cerebral infarction and myocardial infarction. In recent years, statin-based lipid-lowering therapy, blood pressure management, and lifestyle risk changes have greatly reduced the incidence of cardiovascular disease. However, the long-term residual risk still remains. For instance, muscle and skeletal-related symptoms and the associated biochemical perturbations are the most common adverse effects associated with statin use (3).

Reverse cholesterol transport (RCT) is the transport of excess cholesterol from peripheral tissues to the liver, where it is converted into bile acid, and then excreted from the feces. The liver's conversion of cholesterol into bile acid is the primary pathway for cholesterol metabolism, accounting for almost nearly of the cholesterol excreted in the body every day. Boosting RCT and enhancing its conversion into bile acid are the main ways of cholesterol metabolism (4). RCT is an important link in regulating the homeostasis of lipids in the body, with the role of anti-Atherosclerosis and protecting cardiovascular system. Peroxisome proliferator-activated receptors (PPARs) are important regulators in many pathological processes, including plasma lipoprotein balance, foam cell formation, inflammatory response, and plaque stability (5).Research has shown that the PPAR-*γ*/LXR-α pathway could be a potential therapeutic target in regulating the progression of atherosclerosis by promoting the outflow of cholesterol through increased the expression of ABCA1 and ABCG1 (6–8). Therefore, it is crucial to explore novel strategies and agents that promote cholesterol metabolism, in order to optimize the current treatment of atherosclerosis.

The gut microbiota is considered to be an independent and important metabolic organ. The gut microbiota may play an important role in human health by regulating cholesterol metabolism, the release of inflammatory factors, the homeostasis of the flora itself and the immune defence in AS (9). Conversion of cholesterol to bile acids by the liver is the main pathway of cholesterol metabolism. Free bile acids dissolve intestinal lipids and increase hydrophobicity, which helps to reduce intestinal cholesterol absorption and faecal bile acid excretion (10). The microbiota dissociates, dehydroxylates, oxidises and differentially isomerises bound bile acids to free bile acids, and bile salt hydrolases (BSHs) play an important role in this process; BSH-rich enzymes are found in Gut microbiota such as *Clostridium*, *Bacteroides* and *Lactobacillus*.Susan A Joyce's study significantly reduced body weight gain, serum cholesterol [total cholesterol (TC)] and hepatic triglycerides (TG) in conventionally reared mice by increasing the activity of BSH, which was found to be a major contributor to this process (11).

Jiangzhiruanmai Recipe (JZRM), a renowned empirical prescription developed by Professor Ruan Shiyi, a national master of Chinese medicine, is a herbal compound widely applied in clinical practice for the management of cardiovascular diseases. JZRM is formulated from a range of herbal components including *Taxillus sutchuenensis* (Lecomte) Danser (parasitic Loranthus, Sangjisheng), *Poria cocos*(Fuling), *Sargassum pallidum* (Turn.) C. Ag. (seaweed, Haizao), *Ecklonia kurome* Okam. (Laminaria, Kunbu), *Ligusticum striatum* DC. (Ligusticum wallichii, Chuanxiong), and *Polygonum cuspidatum*(Hu zhang).The active components in the JZRM formula, such as pachyman from Poria cocos and effective constituents from Sargassum (including Laminaria), have been demonstrated to inhibit the activation of the TLR4/NF-*κ*B signaling pathway, reduce the expression of inflammatory factors, and synergistically regulate macrophage polarization toward the anti-inflammatory M2 phenotype, thereby suppressing the progression of atherosclerotic plaques (12, 13). In this study, we intend to further investigate the molecular mechanism of JZRM against Atherosclerosis in ApoE^−/−^ mice.

## 2 Materials and methods

### 2.1 Experimental materials

#### 2.1.1 Animals

Seventy-five 8-week-old male ApoE^−/−^ mice and fifteen wild-type C57BL/6J mice weighing (18 ± 2 g) were obtained from SPF (Beijing) Biotechnology Co., Ltd. and the certificate number was SCXK (Beijing) 2016-0006. All animals were maintained under conditions of temperature (20 ± 5 °C), relative humidity (55 ± 5%), alternating lighting (12 h light/12 h dark cycle) and free access to diet. All the experimental protocols were conducted in accordance with the guidelines approved by the Animal Care Committee of Tianjin University of Traditional Chinese Medicine and Animal Ethical Committee of Tianjin University of Traditional Chinese Medicine (TCM–LAEC2021037).

#### 2.1.2 Preparation of medicines

JZRM was provided by Beijing Kangrentang Pharmaceutical Co., Ltd.The main ingredients of JZRM include *Taxillus sutchuenensis* (Lecomte) Danser (parasitic Loranthus, Sangjisheng),*Poria cocos*(Fuling), *Sargassum pallidum* (Turn.) C. Ag. (seaweed, Haizao), *Ecklonia kurome* Okam. (Laminaria, Kunbu), *Ligusticum striatum* DC. (Ligusticum wallichii, Chuanxiong), and *Polygonum cuspidatum*(Hu zhang). Atorvastatin calcium (Batch no. J20070060) was produced by Pfizer Inc. (New York, NY, United States).

#### 2.1.3 Grouping and drug administration

The control group of C57BL/6J mice (Control) was fed a regular mouse chow diet. On the other hand, ApoE-deficient mice (MD12015A, Medicience, China) were fed a high-fat diet to establish an atherosclerosis model. The ApoE-deficient mice were randomly divided into five groups, including a model group (Model), JZRM (4.55 g/kg), JZRM (9.1 g/kg), JZRM (18.2 g/kg), and a positive drug control group (Ator) 1.3 mg/kg, with 15 mice in each group. All animals were given a normal diet for one week before the experiment started. JZRM was administered by gavage, while the vehicle [0.5% carboxymethylcellulose sodium (CMC-Na)] was administered to the Control and Model groups. Each group received a comparable medicine at a dose of 10 mL/kg once per day for 16 weeks.

### 2.2 Identification of bioactive ingredients from JZRM

#### 2.2.1 Sample preparation

100 mg of JZRM extracts was accurately weighed, dissolved in 50% methanol, and vortexed for 30 s. The dissolved JZRM sample solution was sonicated in an ice-cold ultrasonic water bath for 30 min, after centrifugation (12,000 rpm for 15 min), the supernatant collected was filtered using 0.22*μ*m membrane filter, and subsequently, 5 µL was used for LC-MS assessment.

#### 2.2.2 LC-MS analysis

The LC-MS analysis was carried out on a Waters ACQUITY UPLC HSS T3 2.1 × 100mm × 1.8um (LC-147). The column temperature was set at 35℃, with a sample injection volume of 0.2 µL and a flow rate of 0.3 mL/min.The mobile phase consisted of 0.1% formic acid in water (A) and 0.1% formic acid in acetonitrile (B). A multi-step linear elution gradient system was employed: 0 min, 5% A; 27 min, 50% A; 33 min, 90% A; 36 min, 90% A;37 min, 5% A;42 min, 5% A.The sheath gas flow rate was 8L/min, and the sheath capillary temperature was maintained at 250℃. The drying gas flow rate was 6 L/min, with a drying gas temperature at 250℃. Collision energy was set at 10/20/40 eV, while the Cone Voltage was 100 V. Raw data were imported into Qualitative Analysis 10.0 software and the identification process of unknown compounds was established by wizard settings and method templates. Characteristic peaks in the samples were analyzed, extracted molecular ion chromatography peaks and isotope peaks were fitted to possible molecular formulas and matched with PCDL phytoconstituent libraries, and the secondary fragments were matched with PCDL secondary databases, and the results were identified after filtering for quality deviations greater than 7.5 ppm and match scores less than 80. Meanwhile, the online database Sirius version 5.8.5 matched the results of secondary fragments and selected compounds with match scores greater than 80 and FingerID less than 50 to obtain the final report with references.

To characterize functional compounds of JZRM, we performed LC-MS chemical fingerprint profiling using JZRM granula. The positive and negative ion modes of LC-MS were used to generate the chemical base peak intensity chromatogram (BPC) of JZRM extracts. After carefully analyzing the matching results obtained from the SIRIUS 5.8.5 online database for secondary fragments, we have narrowed down our selection criteria to compounds that exhibit a matching score greater than 80 and a FingerID value less than 50. A total of 18 compounds, including Gallic acid, modin 1-glucoside, Hyperoside, Quercitrin and Senkyunolide A, etc. (As shown in Supplementary Figure 1 and Supplementary Table 1).

### 2.3 Quantification of lipid levels and total bile acid (TBA)

All mice were subjected to blood collection from the canthus. Following a 30-minute period of static settling, serum was isolated via centrifugation at 3,500 rpm and 4 °C for 10 min (using a Legend Micro 17 centrifuge, Thermo Scientific, Waltham, MA, USA). The serum samples were then analyzed using an automatic biochemical analyzer (Microlab300, Vertu, Netherlands) to determine the levels of total cholesterol (TC), triglycerides (TG), low-density lipoprotein cholesterol (LDL-C), and high-density lipoprotein cholesterol (HDL-C).

### 2.4 Changes in fecal bile acid composition

Weigh about 50 mg of the sample, add 400 μL of methanol, vortex for 1 min, grind and put it in a centrifuge at 12,000rpm, 4 ℃ for 10 min. Take 50 μL of the supernatant, add 950 μL of 30% methanol, vortex for 30 s, and take 300 μL of the supernatant to filter through a 0.22 μm membrane filter. The filtered liquid is added to the detection bottle. Chromatographic conditions: using ACQUITY UPLC BEH C18 chromatographic column (2.1 × 100 mm, 1.7 μm, Waters, USA), injection volume of 5μL, column temperature of 40℃, mobile phase A-0.01% formic acid water, B-acetonitrile. The gradient elution conditions are as follows: 0–4 min, 25% B; 4–9 min, 25–30% B; 9–14 min, 30–36% B; 14–18 min, 36–38% B; 18–24 min, 38–50% B; 24–32 min, 50–75% B; 32–33 min, 75–90% B; 33–35.5 min, 90–25% B. The flow rate is 0.25 mL/min. Mass spectrometry conditions: electrospray ionization (ESI) source, negative ionization mode. The ion source temperature is 500 ℃, the ion source voltage is −4,500 V, the collision gas is 6 psi, the curtain gas is 30 psi, and the nebulizing gas and auxiliary gas are both 50 psi. Multiple reaction monitoring (MRM) is used for scanning.

### 2.5 16S rRNA sequence

The 16S rRNA sequencing analysis steps can be found in our previously published article (14).The 16S rRNA gene sequencing workflow is detailed in our previously published article. Briefly, colonic contents were collected post-administration and immediately frozen at −80 °C. Total genomic DNA was extracted using a magnetic bead-based method, followed by quality assessment using NanoDrop 2000 and 1% agarose gel electrophoresis. The V3–V4 hypervariable region of the bacterial 16S rRNA gene was amplified using the 338F/806R primer pair in a 20 μL reaction system containing 2.5 mM dNTPs, 0.8 μL Fastpfu DNA polymerase, and 0.4 μL BSA, with an initial denaturation at 95 °C for 2 min, followed by 30–35 cycles of 95 °C for 20 s, annealing at Tm−5 °C for 20 s, and extension at 72 °C for 4 kb/min, and a final extension at 72 °C for 5 min. The amplicons were purified using a gel extraction kit, quantified with a Quantus fluorometer, and pooled according to sequencing depth requirements, and subjected to library preparation using the NEXTFLEX Rapid DNA-Seq Kit. Sequencing was performed on an Illumina MiSeq PE300 platform. Raw reads were quality-filtered with fastp (v0.20.0), assembled using FLASH (v1.2.7), clustered into operational taxonomic units with UPARSE (v7.1) at 97% sequence similarity, and taxonomically annotated against the Silva 16S rRNA database (v138) using RDP classifier (v2.2) with a 70% confidence threshold. following standard bioinformatic pipelines.

### 2.6 Mouse atherosclerotic plaque and collagen content

#### 2.6.1 Aortic gross oil red O staining

Dissect the intact mouse aorta and remove external fat deposits using a microscope (Leica S8 APO, Leica Microsystems, Wetzlar, Germany). Place the aorta in 4% paraformaldehyde solution (P1110, Solarbio, China), then add 70% isopropanol (8018GR0500, Tianjin Xiehe, China) for 5 min. Stain with oil red O staining solution [G1261, Beijing Solarbio (China)] for 15 min, differentiate with 75% alcohol for 1 min, rinse with distilled water and take photos for preservation.

#### 2.6.2 Oil red O staining

The aortic root was fixed, dehydrated, and frozen sectioned (RM 2016, Leica Microsystems). The thickness of the sections was 8μm. The frozen sections were placed in 4% paraformaldehyde solution (P1110, Solarbio, China) for 15 min. After staining with oil red O staining solution for 15 min, the sections were immersed in 60% isopropanol for 30 s, then stained with hematoxylin for 2 min, counterstained with eosin for 5 min, and dried with filter paper. Finally, the sections were mounted with glycerol gelatin. Digital microscopy (Pannoramic MIDI, 3D HIES TECH, Hungary) was used to collect images of the sections, and Image ProPlus 6.0 software to analysis plaque area.

#### 2.6.3 Haematoxylin-eosin staining

Fix the aortic valve tissue in 4% paraformaldehyde, embed it in paraffin, and cut paraffin sections (5μm) that are mounted on slides for hematoxylin-eosin (HE) staining. Deparaffinize the paraffin sections with xylene solution, dehydrate with graded ethanol solution, and stain according to the program of the HE staining kit (G1346, Beijing Solarbio). Digital microscopy (Pannoramic MIDI, 3D HIES TECH, Hungary) was used to collect sections, and Image ProPlus 6.0 software was used for plaque area analysis.

#### 2.6.4 Masson staining

The paraffin sections described above were subjected to Masson staining. Deparaffinize the paraffin sections with xylene solution, dehydrate with graded ethanol solution, and stain according to the program of the Masson staining kit (G1346, Beijing Solarbio). Digital microscopy (Pannoramic MIDI, 3D HIES TECH, Hungary) was used to collect images of the sections.Quantification of collagen content was performed using Image ProPlus 6.0 software. Collagen fibers stained positive with Masson (blue-green regions) were identified and segmented by color thresholding. The percentage of collagen area relative to total plaque area was calculated as follows: % collagen content=(collagen-positive area/total plaque area) × 100%. Three consecutive sections per animal were analyzed, and the average value was taken.

### 2.7 Inflammatory response and fasting blood glucose (FBG)

The levels of TNF-α and IL-6, inflammatory factors in ApoE^−/−^ mice serum were measured with enzyme-linked immunosorbent assays to evaluate the effect of JZRM on the inflammatory response using the specific experimental methods recommended by the manufacturer (Boster Biological Technology, Pleasanton, CA, United States). Color development at 450 nm was then measured using an ELISA autoanalyzer (Enpire, PERKin Elmer, United States). The levels of FBG was measured using an automatic biochemical analyzer (Microlab300, Vertu, Netherlands).

### 2.8 Real-time quantitative PCR analysis

Total RNA was extracted from the liver of ApoE^−/−^ mice using the RNA pure tissue kit (CW0560S, CoWin Biosciences, China). After complete liver lysis and centrifugation, the RNA particles were washed, dried, and quantified (NanoDrop™ One, Thermo Scientific, Waltham, MA, United States). Reverse transcription of RNA was performed using the 5x HiFiScript RTMaster Mix kit (18091200, Cowin Biotech, Jiansu, China) according to the manufacturer's instructions. Amplification was performed using the MagicSYBR mixture according to the manufacturer's instructions (CW0659, CoWin Biosciences), with primers listed in Table 1. The results were analyzed using the 2-*ΔΔ*CT method with GAPDH as an internal control.

**Table 1:** The primer sequences.

Gene	Primer sequences
PPAR*γ*	GCCAAGGTGCTCCAGAAGATGAC
	GGTGAAGGCTCATGTCTGTCTCTG
LXR*α*	AACTGAAGCGGCAAGAAGAGGAAC
	TGGCAGGACTTGAGGAGGTGAG
ABCA1	AGAAGGAGGCTCGGCTGAAGG
	GAGGGATGAGGCTGCTAACAAACC
ABCG1	CATGCTGCTGCCTCACCTCAC
	TCTCGTCTGCCTTCATCCTTCTCC
SRB1	CTCCCAGACATGCTTCCC
	TGCTGAGTCCGTTCCATTT
FXR	GCCAGCGAATCTCACCACAGTAC
	CAGTCAGTCCATCCATCAGAACAGC
CYP7A1	GGAAATACCACGGAAAACAT
	CGCTCTTTGATTTAGGAAGG
GAPDH	GTGCCGCCTGGAGAAAC
	AAGGTGGAAGAGTGGGAGT

### 2.9 Western blot

Mouse liver tissue protein was extracted from the lysate, and after protein quantification, lysate and sample buffer were added proportionally and boiled at 100 °C for 5 min. After the samples were loaded, electrophoresed, transferred to the membrane and incubated with antibodies, the immunoreactive zones were evaluated in the dark using the Enhanced Chemiluminescence) kit and the relative expression levels of the proteins were quantified using ImageJ (Image JUSA). (PPAR*γ*, 1: 2,000, Abcam, ab45036; LXR*α*, 1:500, Abcam, ab176323; ABCA1, 1:1,000, Abcam, ab18180; ABCG1, 1:500, Abcam, ab52617; SRB1, 1:1,000, Abcam, ab52629; FXR, 1:500, Boster, M03308; CYP7A1, 1:1,000, Boster, PB0578; *β*-actin,1:1,000, Abcam, ab8227.)

### 2.10 Statistical and computational methods

The experimental data were recorded in Microsoft Excel and presented as mean ± standard deviation (SD). Statistical analysis was performed using SPSS 21.0 software, Comparisons among multiple groups were conducted using one-way analysis of variance (ANOVA) followed by Tukey's *post-hoc* test for pairwise comparisons. A value of *P* < 0.05 was considered statistically significant. The final data were imported into Graphpad Prism 10.1.2 for graphical representation. Quantification of plaque area and collagen content was performed using Image 1.54P.

## 3 Results

### 3.1 Effect of JZRM on blood lipid levels and TBA

**Figure 1:**
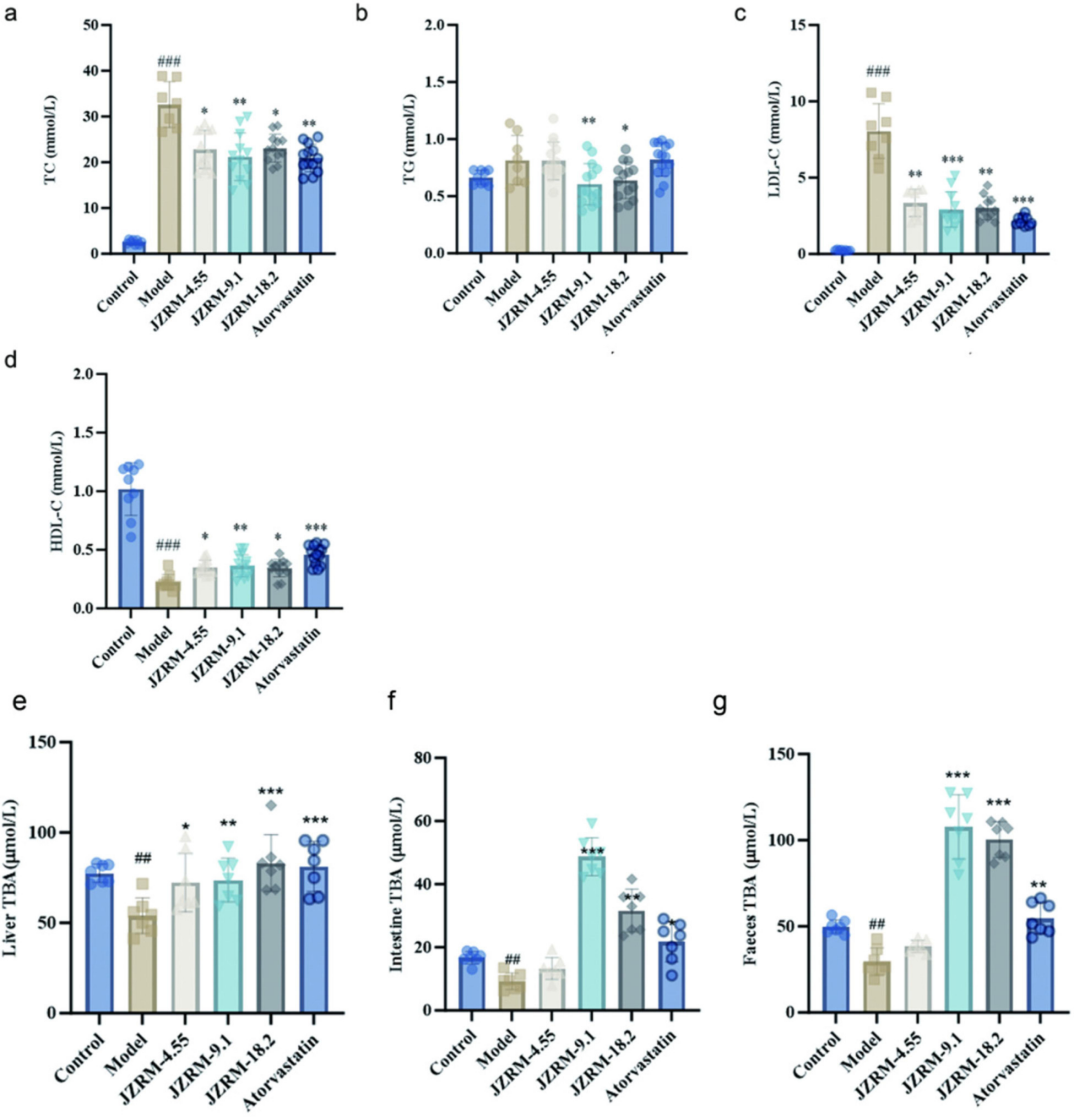
Effect of JZRM on the lipid metabolism and TBA. **(a)** Total cholesterol (TC); **(b)** Low-density lipoprotein cholesterol (LDL-C); **(c)** Triglyceride (TG); **(d)** High-density lipoprotein cholesterol (HDL-C); **(e)** liver total bile acid (TBA); **(f)** intestine TBA; **(g)** faeces TBA after 12 weeks of treatment with JZRM (0.75 or 1.5 g/kg), atorvastatin (2.5 mg/kg), or vehicle. *n* = 10-14. _###_*P* < 0.001 vs. Control, *P < 0.05, **P < 0.01, ***P < 0.001 vs. Model.

Compared with the control group, the TC,TG and LDL-C concentrations in the model group were significantly increased (*P* *<* 0.001, Figures 1a–c), while HDL-C was significantly decreased (*P* *<* 0.001, Figure 1d). Compared with the model group mice, the TC, TG and LDL-C concentrations were significantly reduced (*P* *<* 0.05) in the JZRM 4.55 g/kg, JZRM 9.1 g/kg, JZRM 18.2 g/kg, and Atorvastatin group, the HDL-C concentration was significantly increased (*P* *<* 0.001) (Figure 1b). The liver, ileum, and fecal TBA levels were significantly decreased in the model group (*P* *<* 0.01, Figures 1e–g). Compared with the model group, the TBA levels of liver, intestine and faeces were significantly increase (*P* *<* 0.01, Figures 1e–g) in the JZRM 9.1 g/kg, JZRM 18.2 g/kg, and Atorvastatin groups, indicating that the JZRM can increase the synthesis of bile acid pools and promote the excretion of bile acids through feces.

### 3.2 Effect of JZRM on TNF-α, IL-6, and FBG

**Figure 2:**
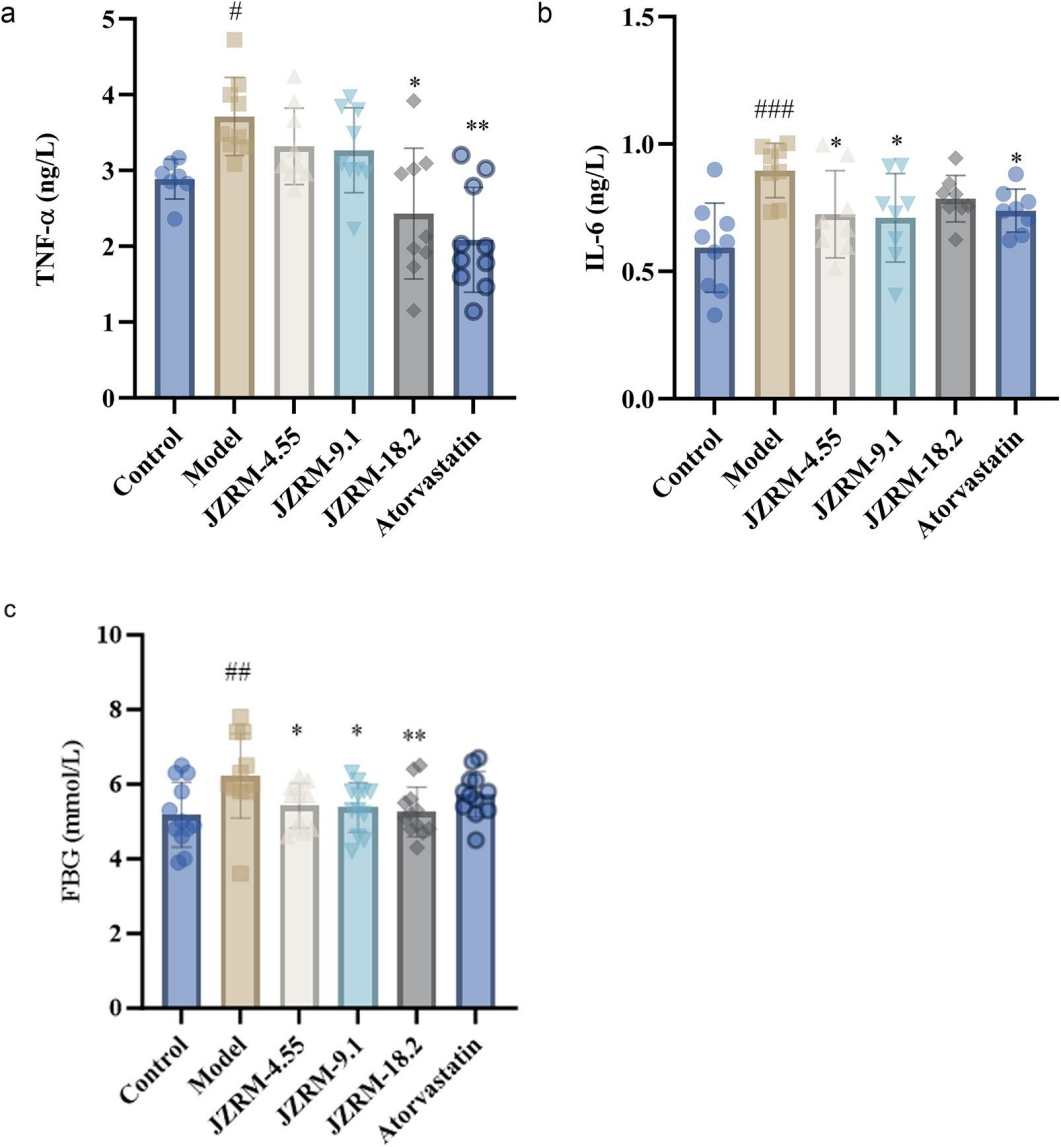
Effect of the JZRM on serum TNF-α, IL-6, and FBG. **(a)**: TNF-α. **(b)**: IL-6. **(c)**: FBG. *n* = 8-10. *^#^P* *<* 0.05, *^##^P* *<* 0.01,*^###^P* *<* 0.001 vs. Control, *P *<* 0.05, **P *<* 0.01 vs. Model.

Compared with the control group, the TNF-α, IL-6 and FBG levels in the model group were significantly increased (*P* *<* 0.05, Figures 2a–c). Compared with the model group, IL-6 levels were reduced (*P* *<* 0.05) in JZRM 4.55 g/kg, JZRM 9.1 g/kg, and Atorvastatin groups, while TNF-α levels were reduced (*P* *<* 0.05) in the JZRM 18.2 g/kg group. FBG levels were reduced (*P* *<* 0.05) in JZRM 4.55 g/kg and JZRM 9.1 g/kg groups, and significantly reduced (*P* *<* 0.01) in JZRM 18.2 g/kg compared with model group.

### 3.3 Effect of the JZRM on vascular pathology

**Figure 3:**
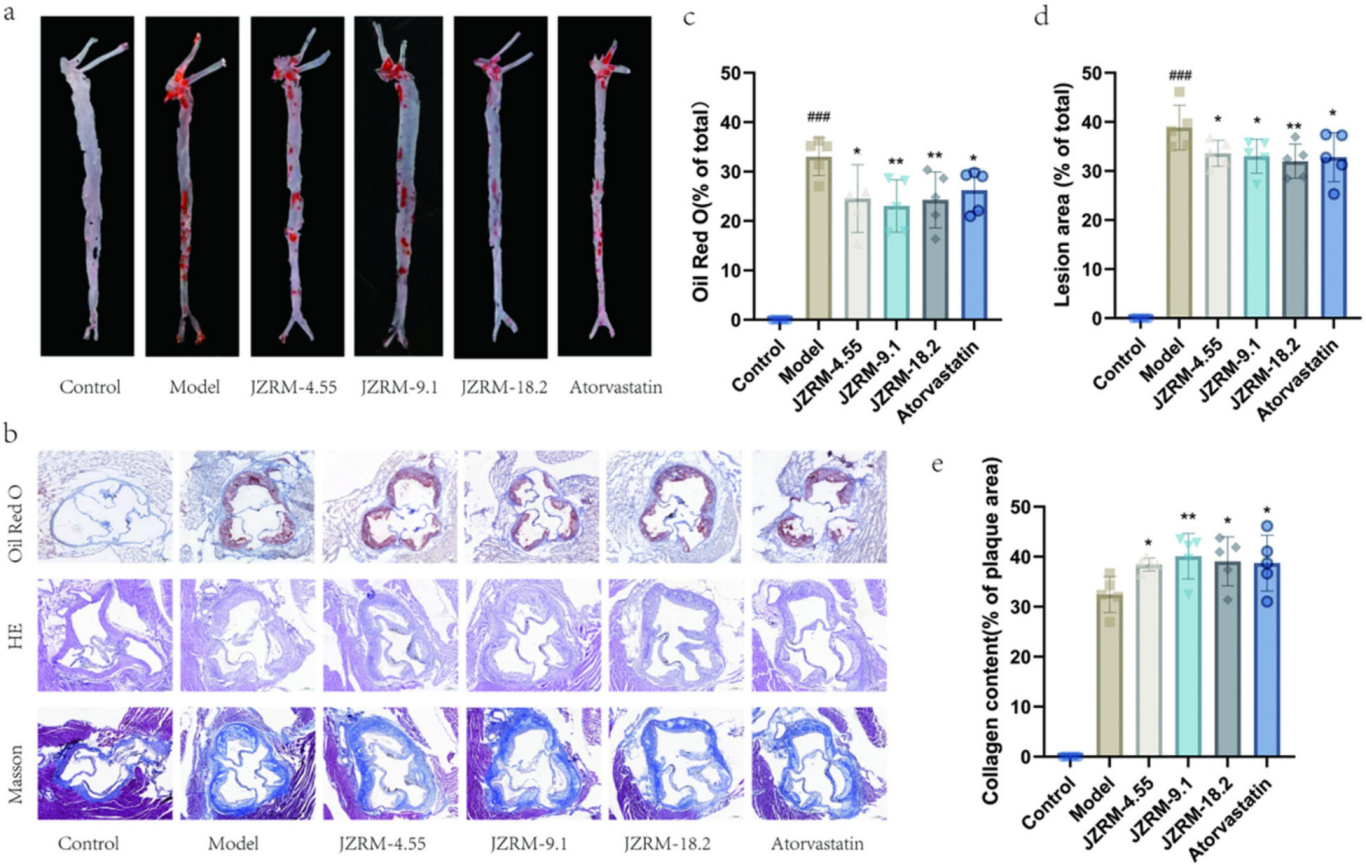
The effect of JZRM on the macroscopic vascular plaques. **(a)**: Gross Oil Red O staining of the aorta, *n* = 3. **(b)**: Oil Red O staining of Aortic root plaque area (×200, 50㎛), Hematoxylin staining of Aortic root plaque area (×200, 50㎛), Masson staining of Aortic root plaque area (×200, 50㎛). **(c)**: Oil Red 0% of total. **(d)**: Lesion area (% of total). **(e)**: Collagen content (% of plaque area). ^##^*P* *<* 0.01, ^###^*P* *<* 0.001 vs. Control, *P *<* 0.05, **P *<* 0.01 vs. Model.

Oil red O staining was used to stain the mouse aorta and aortic valves (Figures 3a, b). Compared with the control group, abundant red plaques were visible in the aortic arch, carotid artery bifurcation, abdominal aorta, and iliac artery of the model group. Compared with the model group, the plaques in the aorta were significantly reduced in the JZRM 4.55 g/kg, JZRM 9.1 g/kg, JZRM 18.2 g/kg, and Atorvastatin groups. Compared with the control group, the intima of the model group was significantly thickened, the blood vessels were significantly narrowed, lipid deposition, red plaques (Figure 3c), plaque area (Figure 3d), and collagen content(Figure 3e) were significantly increased (*P* *<* 0.01). In the JZRM 4.55 g/kg group, the red plaque area was reduced (*P* < 0.05), and the collagen content was significantly increased (*P* < 0.01). In the JZRM 9.1 g/kg group, both the red plaque area and lipid deposition were reduced (*P* < 0.05), and the collagen content was significantly increased (*P* < 0.01). In the JZRM 18.2 g/kg group, the red plaque area and lipid deposition were significantly reduced (*P* < 0.01), and the collagen content was also significantly increased (*P* < 0.05). In the atorvastatin group, lipid deposition was reduced and collagen content was increased (*P* < 0.05). These results indicate that JZRM can reduce plaque area, increase plaque stability, and inhibit atherosclerosis.

### 3.4 The effect of JZRM on the mRNA expression related to RCT

**Figure 4:**
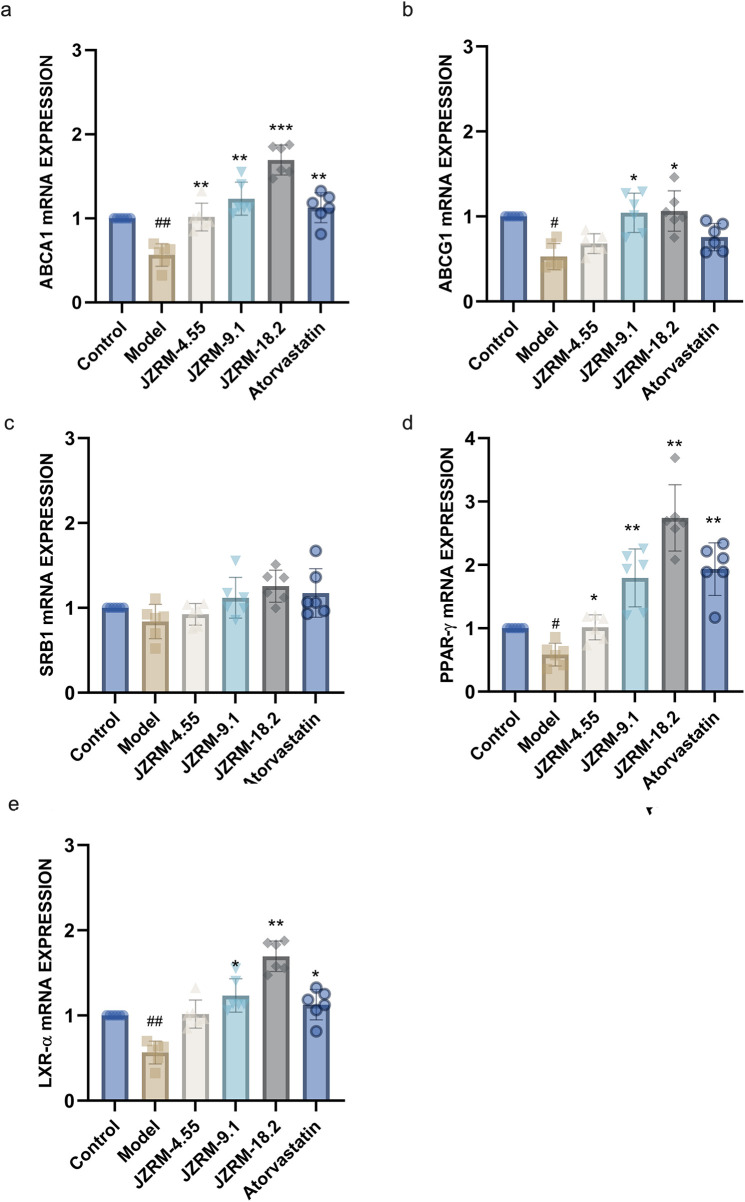
The effect of JZRM on the mRNA expression related to RCT. **(A–E)**: Quantitative analysis of mRNA from RT-PCR. **(a)**: ABCA1, **(b)**: ABCG1, **(c)**: SRBI, **(d)**: PPAR*γ*, **(e)**: LXR-α, *n* = 6. #*P* < 0.05, ##*P* < 0.01vs. Control,*P < 0.05, **P < 0.01, ***P < 0.001vs. Model.

Compared with the control group, the expression of ABCA1, ABCG1, PPAR*γ* and *LXR-ɑ* genes in the liver tissue was significantly decreased in model group (*P* *<* 0.05) (Figures 4a, b, d, e). Compared with the model group, the expression of *ABCG1* and *LXR-ɑ* mRNA was increased in the JZRM 9.1 g/kg and JZRM 18.2 g/kg groups (*P* *<* 0.05). The expression of *ABCA1* and *PPARγ* mRNA was significantly increased in the JZRM 9.1 g/kg, JZRM 18.2 g/kg, and Atorvastatin groups (*P* *<* 0.01). *SRB1* No statistical significance between groups (Figure 4c).

### 3.5 The effect of JZRM on the protein related to RCT

**Figure 5:**
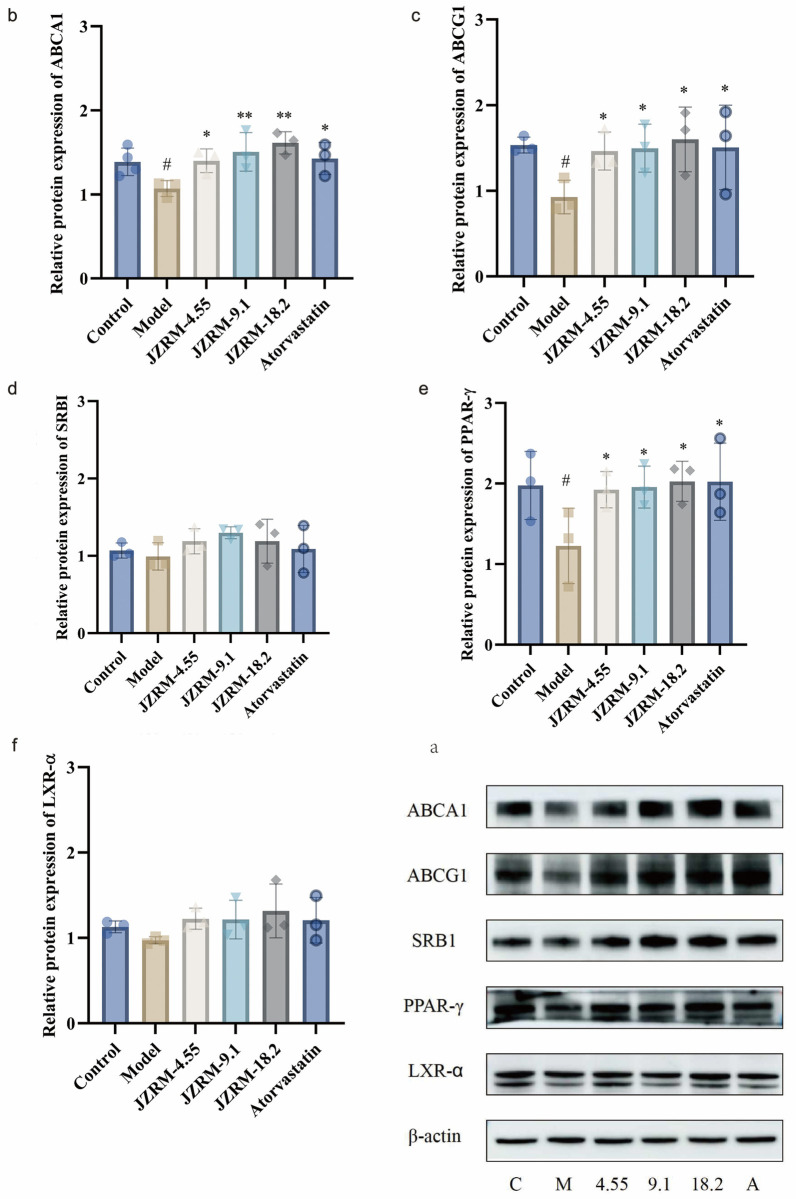
The effect of JZRM on RCT. **(a)**: Western blot of RCT- related proteins in the hepatic. *n* = 3, *β*-actin was used as loading control. **(b)**: ABCA1, **(c)**: ABCG1, **(d)**: SRBI, **(e)**: PPAR*γ*, **(f)**: LXR-α. *n* = 3. #*P* < 0.05 vs. Control, *P < 0.05, **P < 0.01 vs. Model.

Compared with the control group, the protein expression of ABCA1, ABCG1, and PPAR*γ* in the liver tissue of model group were significantly decreased (*P* *<* 0.05) (Figures 5a, b, c, e). Compared with the model group, the protein expression of ABCG1 and PPAR*γ* was increased in the JZRM 4.55 g/kg, JZRM 9.1 g/kg, JZRM 18.2 g/kg, and Atorvastatin group (*P* *<* 0.05). The protein expression of ABCA1 was significantly increased in JZRM 9.1 g/kg and JZRM 18.2 g/kg (*P* *<* 0.01). SRB1 No statistical significance between groups (Figure 5d).

### 3.6 The effect of JZRM on the protein and mRNA expression of hepatic FXR and CYP7A1

**Figure 6:**
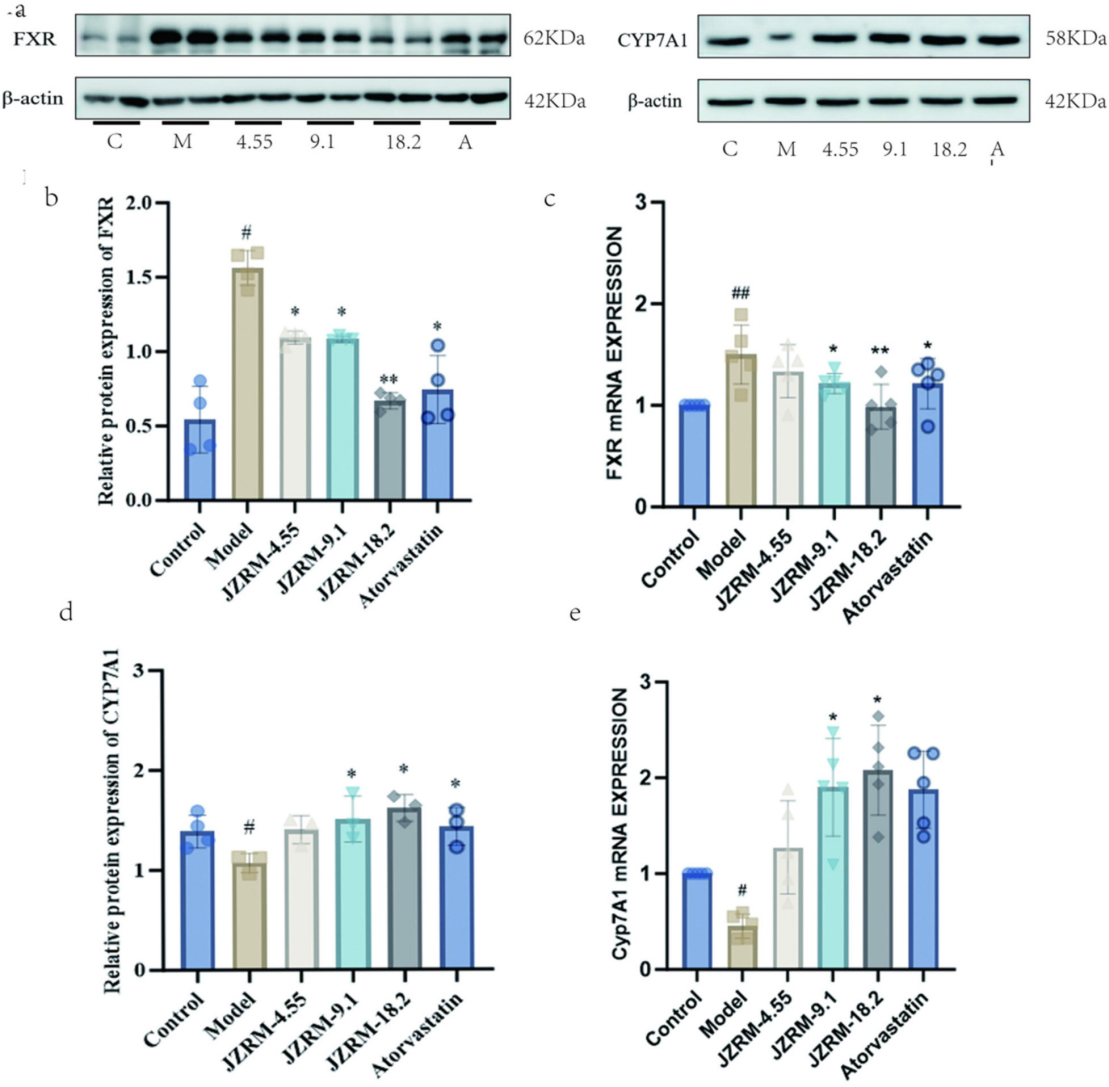
Effect of JZRM on the mRNA and protein expression of hepatic FXR and CYP7A1. **(a,b,d)**: Quantitative analysis of gray values of bands from Western blots, *n* = 3, *β*-actin was used as loading control. **(c,e)**: Quantitative analysis of mRNA from RT-PCR. *n* = 5, **(b)**: FXR, **(c)**: FXR mRNA, **(d)**: CYP7A1, **(e)**: CYP7A1 mRNA. #*P* < 0.05, ##*P* < 0.01 vs. Control, *P < 0.05, **P < 0.01 vs. Model.

Compared with the control group, the protein (Figures 6a, b) and gene expression (Figure 6c) of *FXR* in model group was significantly increased (*P* *<* 0.05), while the protein (Figure 6d) and gene expression (Figure 6e) of *CYP7A1* was significantly decreased (*P* *<* 0.05). Compared with the model group, the protein and gene expression of FXR were decreased in JZRM 9.1 g/kg group, JZRM 18.2 g/kg group and Atorvastatin group (*P* *<* 0.05), while the protein and gene expression of *CYP7A1* were significantly increased in the JZRM 9.1 g/kg group and JZRM 18.2 g/kg group(*P* *<* 0.05).

### 3.7 The effect of JZRM on the composition of bile acids

**Figure 7:**
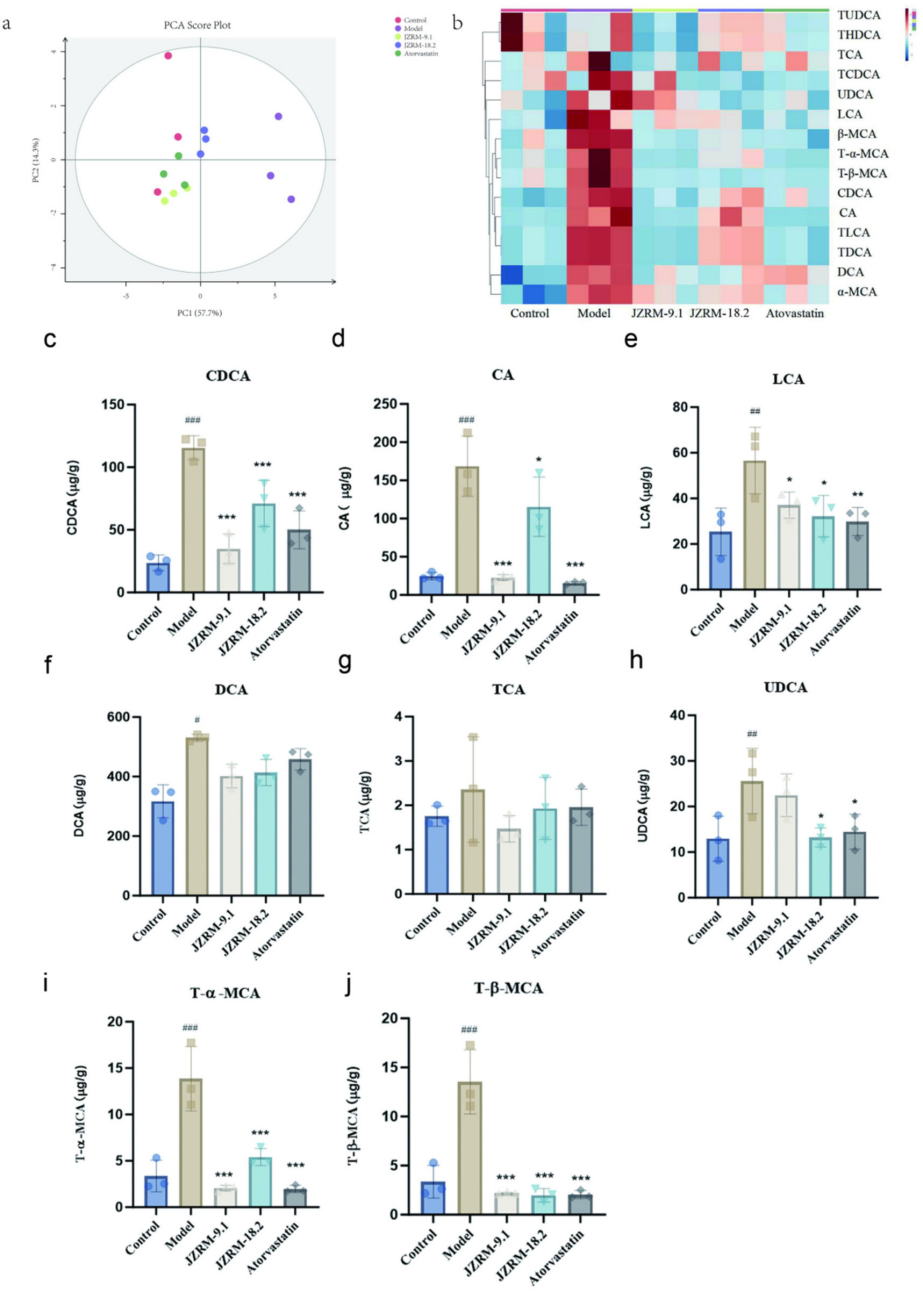
The effect of JZRM on the composition of bile acids in mouse feces. **(a)**: PCA model, **(b)**: Heatmap of bile acids in mouse feces, **(c)**: CDCA, **(d)**: CA, **(e)**: LCA, **(f)**: DCA, **(g)**: TCA, **(h)**: UDCA, **(i)**: T-α-MCA, **(j)**: T-*β*-MCA. *n* = 3. ^##^*P* *<* 0.01, ^###^*P* *<* 0.001 vs. Control, *P *<* 0.05, **P *<* 0.01, ***P *<* 0.001 vs. Model.

LC/MS technology was used to detect the bile acid components in mouse feces. The PCA model reflected the original state of the metabolic data (Figure 7a). Compared with the control group, the primary bile acids CDCA, CA, DCA, and UDCA were significantly increased in the model group (Figures 7b,c, d, f, h, *P* *<* 0.05). Compared with the model group, JZRM 9.1 g/kg significantly reduced the levels of CDCA (*P* < 0.001, Figure 7c) and LCA (*P* *<* 0.05, Figure 7e). JZRM 18.2 g/kg significantly reduced the levels of CDCA (*P* *<* 0.001, Figure 7), LCA (*P* *<* 0.05, Figure 7e), and UDCA (*P* *<* 0.05, Figure 7h). Atorvastatin significantly reduced the levels of CDCA (*P* *<* 0.001, Figure 7c), LCA (*P* *<* 0.01, Figure 7e), and UDCA (*P* *<* 0.05, Figure 7h). TCA, T-a -MCA, T-*β*-MCA No statistical significance between groups (Figures 7g–i, j).

### 3.8 The effect of JZRM on gut microbiota of AS mice

**Figure 8:**
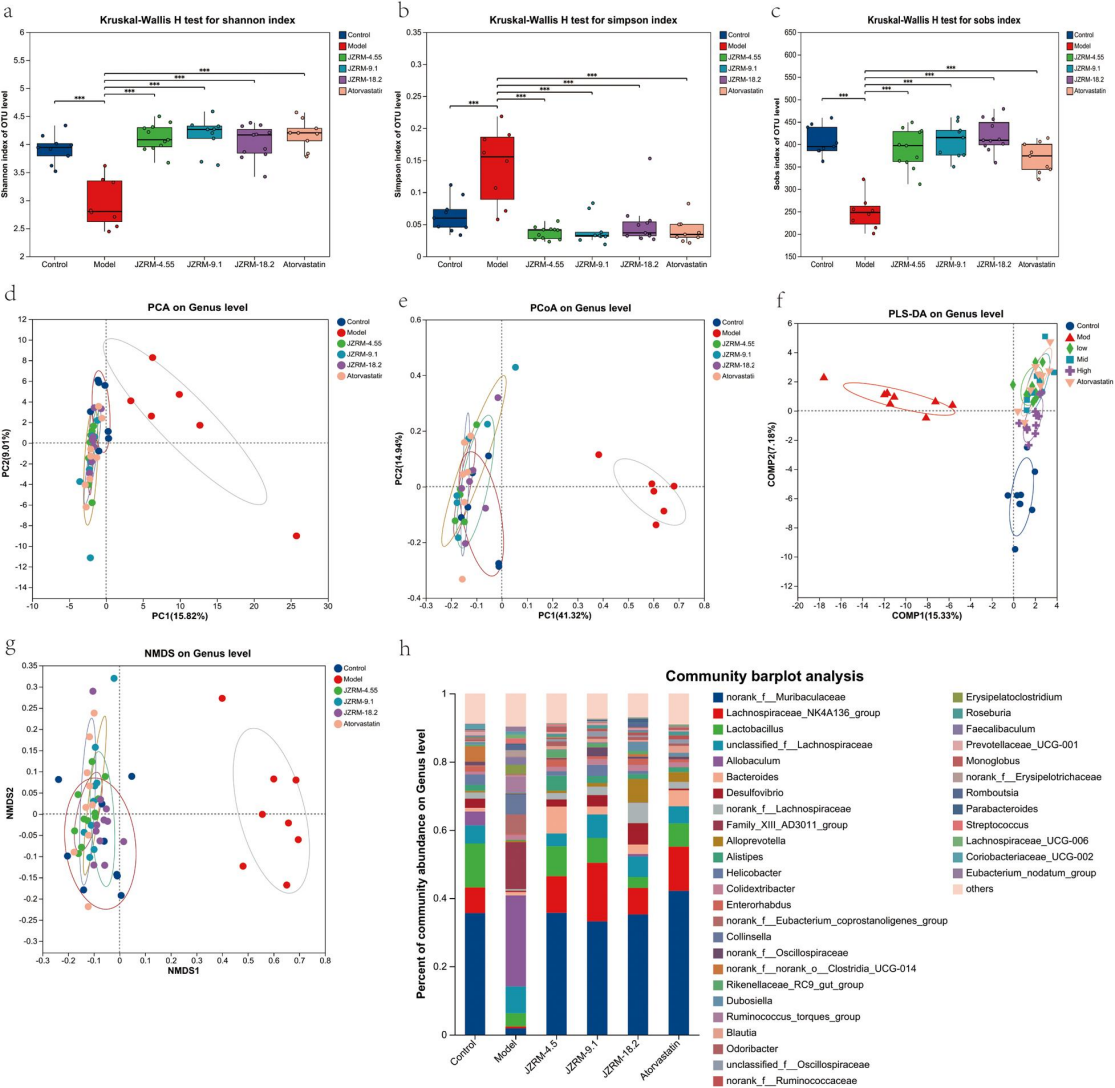
The effect of JZRM on gut microbiota of aS mice. **(a)**: Shannon; **(b)**: Simpson; **(c)**: sobs; **(d)**: PCA; **(e)**: PCoA; f: PLS-DA; **(g)**: NMDS; **(f)**: community abundance on geuns level.

The actual observed value of sobs richness, the larger the value, the higher the community species richness (diversity); Shannon diversity index, the larger the value, the higher the community diversity; Simpson diversity index, the smaller the value, the higher the community diversity. Compared to the control group, the Shannon and Sobs indices significantly decreased and the Simpson index significantly increased in the model group, indicating that the richness and diversity of the colony in the model group significantly decreased; compared to the model group, JZRM (4.55, 9.1, 18.2) significantly increased the Shannon and Sobs indices and decreased the Simpson index, indicating that JZRM was able to increase the richness and diversity of the colony (Figures 8a–c).

We further analysed the differences between the groups at the genus level by principal component analysis (PCA), principal co-ordinates analysis (PCoA), partial least squares discriminant analysis (PLS-DA), non-metric multidimensional scaling (NMDS) to analyse the variability between the groups at the genus level, and the results showed that the model was significantly different from the other groups (Figures 8d–g).

Finally, we observed the changes of bacterial species in each group at the genus level: compared with the control group, the model group norank_f__Muribaculaceae, Lachnospiraceae_NK4A136_group, *Lactobacillus, Bacteroides* levels were significantly; compared with the model group, JZRM (4. 55, 9.1, 18.2 g/kg) norank_f__Muribaculaceae, Lachnospiraceae_NK4A136_group, Lactobacillus group, *Lactobacillus* and *Bacteroides* levels were significantly increased (Figure 8h).

## 4 Disscussion

Cholesterol metabolism represents a dynamic homeostatic process: dietary cholesterol is absorbed in the intestine and transported to the liver, where it is packaged with triglycerides into very low-density lipoprotein (VLDL) for secretion into the circulation. Upon lipolysis during systemic circulation, VLDL is converted to low-density lipoprotein (LDL), which is subsequently taken up by peripheral cells. LDL serves as the predominant source of cholesterol within atherosclerotic plaques, and its excessive deposition directly promotes plaque formation (15). Conversely, peripheral cells efflux surplus cholesterol to high-density lipoprotein (HDL) via ATP-binding cassette transporters and related mechanisms; this cholesterol is then transported back to the liver through reverse cholesterol transport (RCT) for metabolic conversion into bile acids and subsequent excretion, thereby preventing intracellular cholesterol accumulation (16–19).Thus, cholesterol exerts a dual role in the pathogenesis of atherosclerosis—whereas elevated LDL-C levels constitute a pathogenic risk factor, HDL-C confers vascular protection through RCT. Maintaining this metabolic equilibrium, characterized by the suppression of LDL deposition concurrent with the promotion of RCT-mediated cholesterol clearance, represents a critical mechanistic strategy for the prevention and treatment of atherosclerosis.In this study, intervention with JZRM and Ator significantly reduced serum levels of total cholesterol (TC), triglycerides (TG), and low-density lipoprotein cholesterol (LDL-C), while increasing high-density lipoprotein cholesterol (HDL-C). Concurrently, aortic plaque formation was attenuated and collagen content was decreased, suggesting that they may exert anti-atherosclerotic effects by regulating cholesterol metabolic homeostasis and reducing lipid deposition.

The gut microbiota and its metabolites are key exogenous regulatory factors in the progression of atherosclerosis. Microbial diversity directly influences host metabolic phenotypes: high diversity facilitates the production of protective metabolites such as short-chain fatty acids, thereby attenuating inflammatory responses; conversely, reduced diversity leads to metabolic imbalance, activates immune-inflammatory pathways, and promotes atherosclerosis (20–23). Clinical studies have demonstrated that the abundance of beneficial bacteria such as Bacteroides is decreased in the intestines of coronary heart disease patients, accompanied by elevated lipopolysaccharide levels (24). Furthermore, gut microbiota abundance is closely associated with the blood lipid profile; certain bacterial communities promote bile acid excretion via bile acid synthase activity, accelerate hepatic cholesterol conversion, and consequently reduce serum cholesterol levels (25–27). Probiotic intervention (*Lactobacillus plantarum and Lactobacillus rhamnosus*) has also been shown to delay the progression of atherosclerosis (28). The results of the present study indicate that JZRM significantly improved gut microbiota diversity indices and modulated specific bacterial taxa by increasing the abundance of beneficial bacteria (e.g., Muribaculaceae family, *Lachnospiraceae*_*NK4A136_group* genus), thereby reversing the microbial dysbiosis observed in the model group. Notably, although *Allobaculum* has been widely reported as a beneficial genus associated with short-chain fatty acid production and metabolic health, its abundance was increased in the model group and decreased following JZRM treatment in the present study. This discrepancy suggests that the role of *Allobaculum* may be influenced by the disease context. It is possible that the elevation of *Allobaculum* in the model group reflects a compensatory response to gut microbial imbalance and inflammation, rather than a direct indicator of a healthy state. Moreover, given that 16S rRNA sequencing provides resolution primarily at the genus level, functional heterogeneity among different *Allobaculum* species cannot be excluded. Therefore, the decrease in *Allobaculum* after treatment may be associated with the restoration of microbial homeostasis following JZRM intervention.

Reverse cholesterol transport (RCT) plays a pivotal role in the progression of atherosclerosis. High-density lipoprotein (HDL) serves as the primary cholesterol efflux carrier, and its function depends on apolipoprotein A-I (ApoA-I)-mediated cholesterol acceptance and transport. Cholesterol in foam cells is mainly effluxed to HDL through pathways such as ABCA1, ABCG1, and SR-BI, and is subsequently transported to the liver for clearance via multiple enzymatic reactions (29–32). ABCA1 mediates the formation of nascent HDL and dominates macrophage cholesterol efflux, whereas ABCG1 promotes the further transfer of cholesterol to mature HDL; deficiency in either transporter is associated with impaired cholesterol efflux and increased atherosclerotic risk (33–35). SR-BI enhances RCT through selective uptake of HDL cholesteryl esters, and its deficiency exacerbates high-fat diet-induced metabolic disorders and atherosclerotic lesions (36). Collectively, these findings indicate that maintaining the integrity of cholesterol efflux pathways is essential for suppressing foam cell formation and plaque progression.

Peroxisome proliferator-activated receptor gamma (PPAR*γ*), a critical nuclear receptor, plays a central role in regulating lipid metabolism, inflammatory responses, and vascular homeostasis. Previous studies have demonstrated that PPAR*γ* activation not only ameliorates dyslipidemia but also suppresses inflammatory reactions, reduces monocyte recruitment, and enhances plaque stability (37–39). Furthermore, PPAR*γ*upregulates endothelial nitric oxide synthase (eNOS) expression, thereby increasing nitric oxide (NO) bioavailability and exerting vascular endothelial protective effects. Liver X receptor alpha (LXR*α*), an important downstream target of PPAR*γ*, is crucial in cholesterol homeostasis regulation. LXR*α* can form a heterodimer with the retinoid X receptor, bind to the promoter region of ABCA1, and promote the expression of ABCA1 and ABCG1, thereby enhancing cholesterol efflux and inhibiting intracellular lipid accumulation (40–42). Meanwhile, the PPAR*γ*/LXR*α* axis also exerts anti-inflammatory effects by suppressing monocyte differentiation into foam cells and reducing their deposition in the vascular wall (43, 44).

**Figure 9:**
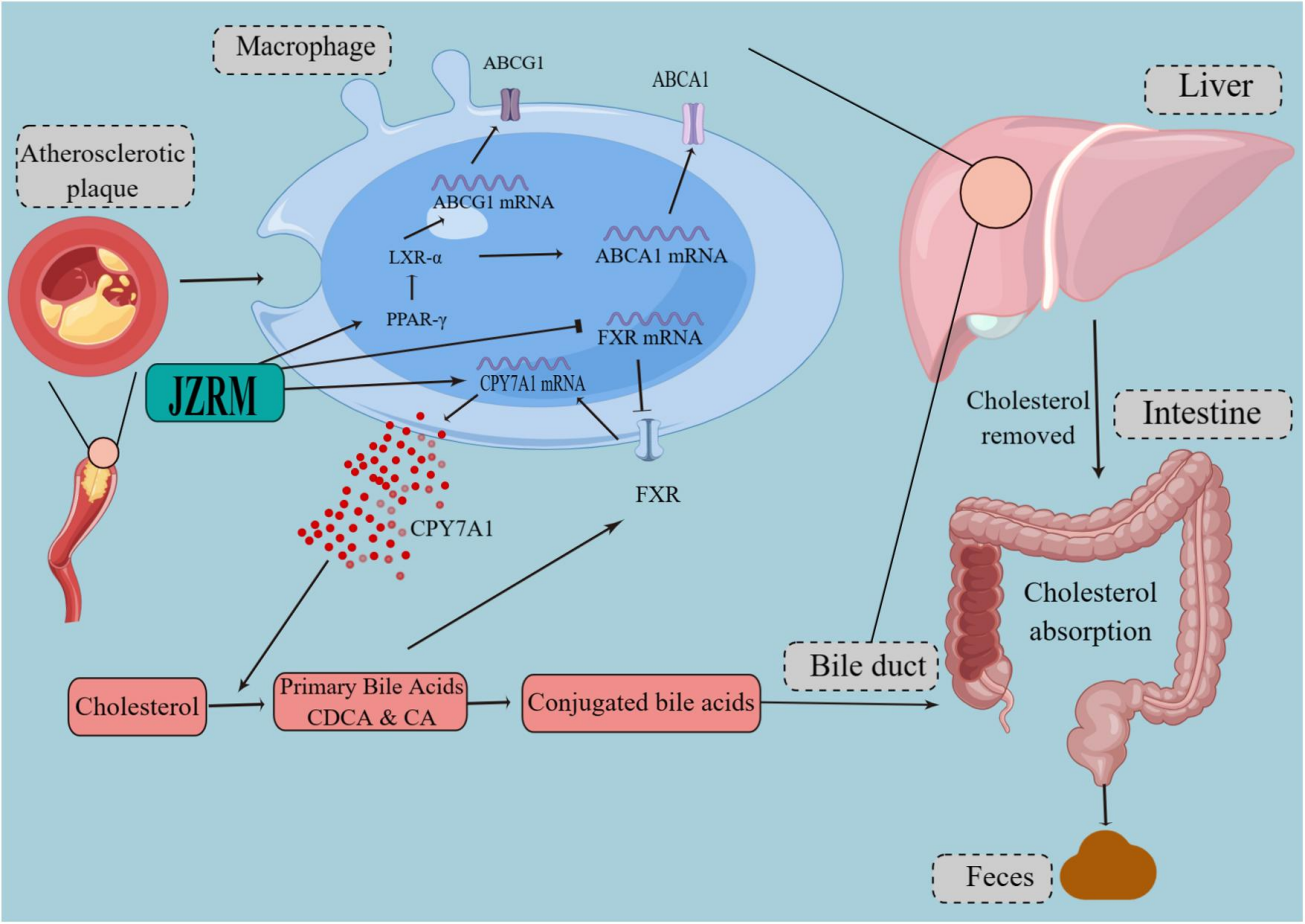
A schematic representation of the identified mechanism of action of RCT in JZRM treated mice. (By Figdraw).

In the present study, compared with the model group, JZRM significantly reduced serum levels of TNF-α and IL-6 in atherosclerotic mice, suggesting its anti-inflammatory properties. Meanwhile, the mRNA and protein expression of PPAR*γ*, LXR*α*, ABCA1, ABCG1, and SR-BI in the liver were all downregulated in the model group, whereas JZRM intervention significantly upregulated the expression of PPAR*γ*, LXR*α*, ABCA1, and ABCG1, and showed a tendency to increase SR-BI expression (Figure 9). These alterations are consistent with the protective characteristics of enhanced RCT and attenuated inflammatory responses.Combined with previous studies, the PPAR*γ*/LXR*α* signaling pathway not only promotes cholesterol efflux through transcriptional regulation of ABCA1 and ABCG1 but also participates in inflammatory response modulation, thereby exerting dual protective effects in atherosclerosis. Therefore, the results of this study suggest that the anti-atherosclerotic effect of JZRM may be associated with its regulation of the PPAR*γ*/LXR*α*-related signaling pathway, improvement of cholesterol efflux, and suppression of inflammatory responses.

Furthermore, cholesterol cleared by the liver must ultimately be converted into bile acids and excreted via the biliary tract, thereby completing the terminal step of cholesterol metabolism.In humans, about 500 mg of cholesterol is converted into bile acids in the liver every day. The main rate-limiting enzyme for bile acid synthesis is Cholesterol 7-alpha-hydroxylase (CYP7A1), and the alternative pathway is Cholesterol 27-alpha-hydroxylase (CYP27A1). Bile acids, including cholic acid (CA) or chenodeoxycholic acid (CDCA), are released into the small intestine after binding with taurine (t) or glycine (g) and are reabsorbed before being recirculated to the liver along with portal vein blood, with over 95% of bile acids being retained. CA and CDCA can be converted into deoxycholic acid (DCA) and lithocholic acid (LCA), respectively, both of which are secondary free bile acids. A small amount of primary bile acids reach the distal ileum and colon, and bile acids bound by microbial bile salt hydrolase (BSH) can be further metabolized into secondary bile acids through dehydrogenation, dehydroxylation, and epimerization by colonic bacteria (45). Recirculating bile acids are secreted into bile again after meals, and a small portion of bile acids escape intestinal absorption and are secreted into feces. This constitutes the main physiological way of eliminating cholesterol from the body since free bile acids dissolve intestinal lipids and increase hydrophobicity, reducing intestinal cholesterol absorption, with most bile acids being excreted in feces. Bile acids are mainly generated from cholesterol under the action of CYP7A1, the main rate-limiting enzyme for bile acid synthesis. A decrease in CYP7A1 expression can lead to a reduction in bile acid synthesis and secretion, thereby promoting the occurrence and development of Atherosclerosis. Conversely, inducing CYP7A1 expression can significantly increase the bile acid pool size, thereby reducing plaque formation in Atherosclerosis (46, 47). In addition, bile acids can act as signaling molecules to activate various nuclear receptors and regulate their own metabolism through downstream effects of nuclear receptor activation, including the farnesoid X receptor (FXR) and the G protein-coupled bile acid receptor TGR5, with FXR being a key nuclear receptor regulating lipid metabolism (48). FXR regulates lipid metabolism by adjusting liver lipid generation pathways, lipid secretion, plasma lipid clearance, and intestinal cholesterol absorption (49). In the liver, FXR activates the transcription of small heterodimer partner (SHP) to replace recombinant liver receptor homolog 1 (LRH-1) to promote the blockade of CYP7A1 transcription (50).

Changes in bile acid composition may be reflected in the level of FXR signaling activation. CDCA is the most effective endogenous ligand for FXR, while TCA, DCA, and CA are moderate FXR agonists, and T*α*MCA and T*β*MCA are effective FXR antagonists (51). In addition, DCA and LCA are two highly hydrophobic bile acids with greater cell toxicity as their hydrophobicity increases. The accumulation of hydrophobic bile acids in the liver is an important cause of cholestasis-induced liver injury (52). By promoting excessive cholesterol conversion into bile acids in the liver, a unique effect on reducing atherosclerotic lesions has been shown, with animal studies demonstrating that ApoE^−/−^ mice fed a high-fat diet for 16 weeks showed FXR inhibition and extensive plaque area. In contrast, inhibition of FXR activation and upregulation of CYP7A1 expression reduced atherosclerotic lesions by 78% and decreased serum low-density lipoprotein cholesterol (LDL-C) levels by 73.9% (52). The results revealed that JZRM inhibited the protein and mRNA expression of FXR and promoted the protein and mRNA levels of CYP7A1. Furthermore, LC/MS was utilized to examine the content of bile acid spectrum in mouse feces, and JZRM significantly reduced the expression of FXR effective endogenous agonists CDCA and LCA, moderately reduced the level of moderate FXR agonists TCA, DCA, and CA, but had no significant effect on FXR agonists T-α-MCA and T-*β*-MCA. The above results first demonstrate that JZRM can reduce the levels of bile acids such as CDCA, LCA, TCA, DCA, and CA, inhibit FXR expression, upregulate CYP7A1 levels, promote cholesterol to synthesize more bile acids in the liver and enter the intestine for excretion through feces, thereby achieving a protective effect on ApoE^−/−^ mice.

In terms of clinical translation, our animal experiments verified that JZRM could remodel gut microbiota structure and correct abnormal bile acid metabolism, offering a new mechanistic explanation for its pharmacological effect. As a multi-target herbal formula, JZRM has favorable safety profiles and broad clinical application prospects. However, preclinical data cannot fully represent human responses. Future large-animal studies and standardized clinical trials are essential to optimize dosage, confirm efficacy and safety, and promote the clinical transformation of this formula.

### 4.1 Limitations

The present study only analyzed the changes in gut microbiota in ApoE^−/−^ mice regulated by JZRM, but did not investigate the causal role of the microbiota in the therapeutic effects. No functional intervention experiments (e.g., antibiotic treatment, fecal microbiota transplantation, or germ-free models) were performed. Therefore, the extent to which gut microbiota contributes to the lipid-lowering and anti-atherosclerotic actions of JZRM remains unclear. Future studies should inhibit or deplete gut bacteria to observe the effects of JZRM in ApoE⁻/⁻ mice, thereby clarifying the involvement of the microbiota. Furthermore, the role of key bacterial taxa (e.g., Muribaculaceae) in cholesterol and bile acid metabolism should be investigated.

## 5 Conclusion

Our research has demonstrated that JZRM can alleviate the reduction of blood lipids, blood glucose levels, inflammatory state, and promote bile acid synthesis. JZRM inhibited Atherosclerosis via modulating PPAR*γ*/LXR*α*/ABCA1/ABCG1 pathway and FXR/CYP7A1, which ultimately affects RCT, and increasing the abundance and diversity of intestinal flora and increased the level of bile salt hydrolases (*Lactobacillus, Bacteroides*) in mice. All in all, JZRM regulates gut microbiota-liver-cholesterol axis, promotes cholesterol synthesis and metabolism to alleviate atherosclerosis. These findings provide a novel mechanism of JZRM in the prevention and reversal of Atherosclerosis.

## Data Availability

The raw data supporting the conclusions of this article will be made available by the authors, without undue reservation.
