# Supplementary material for: Jiangzhi ruanmai recipe alleviates atherosclerosis in ApoE−/− mice by regulating cholesterol metabolism involved in gut microbiota remodeling
Source: Front Cardiovasc Med. 2026 May 29;13:1834983. doi: 10.3389/fcvm.2026.1834983 (PMC13259839; doi:10.3389/fcvm.2026.1834983)
Supplement: Supplementary file 1 [file Table1.docx]

Supplementary Table 1 JZRM Bioactive Ingredients

| number | Name | CAS |
| --- | --- | --- |
| 1 | Gallic acid | 149-91-7 |
| 2 | 3-Furancarboxylic Acid Methyl Ester/Methyl pyromucate | 13129-23-2/611-13-2 |
| 3 | 3,4-Dihydroxybenzoic acid | 99-50-3 |
| 4 | - | - |
| 5 | (+)-Catechin/EC;Epicatechin | 154-23-4/490-46-0 |
| 6 | Polydatin/trans-Resveratrol 4'-o-beta-d-glucuronide | 27208-80-6/38963-95-0 |
| 7 | Hyperoside | 482-36-0 |
| 8 | Guaijaverin | 22255-13-6 |
| 9 | Quercitrin | 522-12-3 |
| 10 | - | - |
| 11 | modin 1-glucoside | 38840-23-2 |
| 12 | - | - |
| 13 | Physcion 1-O-β-D-glucoside | 26296-54-8 |
| 14 | - | - |
| 15 | Senkyunolide A | 63038-10-8 |
| 16 | Dibutyl phthalate | 84-74-2 |
| 17 | - | - |
| 18 | Erucamide/Bis(2-ethylhexyl) phthalate | 112-84-5/117-81-7 |
